# Editorial: Fifty Campbell systematic reviews relevant to the policy response to COVID‐19

**DOI:** 10.1002/cl2.1107

**Published:** 2020-08-10

**Authors:** Ariel Aloe, Eric Barends, Douglas Besharov, Zulfiqar Bhutta, Xinsheng ‘Cindy’ Cai, Marie Gaarder, Ruth Garside, Neal Haddaway, Elizabeth Kristjansson, Brandy Maynard, Lorraine Mazerolle, Robyn Mildon, Sarah Miller, Jan Minx, Peter Neyroud, Annette O'Connor, Denise Rousseau, Ashrita Saran, Joann Starks, Gavin Stewart, Jo Thompson Coon, Peter Tugwell, Jeffrey Valentine, Vivian Welch, Oliver Wendt, Howard White

**Affiliations:** ^1^ Co‐chair Campbell Methods, University of Iowa Iowa City USA; ^2^ Co‐chair Campbell Business and Management, Centre for Evidence Based Management (CEBMa) Leiden The Netherlands; ^3^ Co‐chair Campbell Social Welfare University of Maryland College Park USA; ^4^ Co‐chair Campbell Nutrition Sub‐group University of Toronto Toronto Canada; ^5^ Co‐chair Campbell Knowledge Translation and Implementation American Institutes for Research (AIR) Washington, D.C. USA; ^6^ Co‐chair Campbell International Development International Initiative for Impact Evaluation (3ie) London UK; ^7^ Co‐chair Campbell Methods University of Exeter Truro UK; ^8^ Co‐chair Campbell Climate Solutions Stockholm Environment Institute Stockholm Sweden; ^9^ Co‐chair Campbell Nutrition Sub‐group University of Ottawa Ottawa Canada; ^10^ Co‐chair Campbell Social Welfare Saint Louis University St Louis USA; ^11^ Co‐chair Campbell Crime and Justice University of Queensland Brisbane Australia; ^12^ Co‐chair Campbell Knowledge Translation and Implementation Centre for Evidence and Implementation (CEI) Melbourne Australia; ^13^ Co‐chair Campbell Education Queen's University Belfast Belfast UK; ^14^ Co‐chair Campbell Climate Solutions Mercator Research Institute on Global Commons and Climate Change Berlin Germany; ^15^ Co‐chair Campbell Crime and Justice, Institute of Criminology Cambridge University Cambridge UK; ^16^ Co‐chair Campbell Food Security Sub‐group Iowa State University Ames USA; ^17^ Co‐chair Campbell Business and Management Carnegie Mellon University Pittsburgh USA; ^18^ Acting Director Campbell Collaboration South Asia New Delhi India; ^19^ Co‐chair Campbell Disability American Institutes for Research (AIR) Washington, D.C. USA; ^20^ Co‐chair Campbell Food Security Sub‐group Newcastle University Newcastle upon Tyne UK; ^21^ Co‐chair Campbell Ageing Sub‐group University of Exeter Truro UK; ^22^ Co‐chair Campbell International Development, Department of Medicine, Faculty of Medicine University of Ottawa Ottawa Canada; ^23^ Co‐chair Campbell Training University of Louisville Louisville USA; ^24^ Editor in chief, Campbell Collaboration Bruyère Research Institute Ottawa Canada; ^25^ Co‐chair Campbell Disability University of Central Florida Orlando USA; ^26^ CEO, Campbell Collaboration New Delhi India

The global severe acute respiratory syndrome coronavirus 2 pandemic strikingly shows the need for rigorous evidence to inform decisions. During such times of crisis, many decisions are made across multiple sectors and trillions of dollars are spent to deal with its consequences that affect all aspects of economic and societal life. Given the scale of human suffering, thoughtfully designing effective policies, and carefully spending scarce resources on interventions that work during crisis management and recovery, become crucial.

However, in many areas of decision making, the use of robust and reliable evidence is not the norm. This has dire consequences: evidence from impact evaluations in different sectors show that about 80% of policy interventions are not effective (White, 2019). Equally, the reliance on an individual study or model rather than evidence synthesis commonly leads to misinformed policy and outright harm. For example, the retracted study on hydroxychloroquine for COVID‐19 led to public harm as well as public mistrust (Mehra, Ruschitzka, & Patel, 2020).

Now, more than ever, public policy needs to be informed by the most rigorous, comprehensive and up‐to‐date evidence possible. We, at the Campbell Collaboration, are working on both providing this rigorous evidence and promoting its use to inform decisions about social and public policy. Campbell systematic reviews provide a wealth of rigorous evidence to support social and economic response. These reviews highlight what is known and actionable, and point to critical questions decisionmakers need to ask in planning and implementing social and economic responses.

Campbell systematic reviews follow carefully structured, peer‐reviewed procedures to produce high‐quality, theory‐based evaluations of social and economic policies and programmes. They address real‐world problems, often in partnership with relevant stakeholders, and seek to answer what works, why and for whom. Our 12 coordinating groups provide broad coverage of social issues, including ageing, business and management, climate solutions, crime and justice, disability, education, international development, knowledge translation and implementation, methods, nutrition and food systems and social welfare. And our international editorial board supervises the process in order to produce rigorous evidence syntheses and strategic partnerships that encourage their timely consideration for policy.

Campbell systematic reviews have influenced national policy discussions on over 40 topics. They inform international guidelines and support the design and scaling‐up of dozens of evidence‐based social and economic policies and programmes (Campbell Collaboration, 2020).

Campbell also publishes evidence and gap maps, which provide a thorough overview of the body of evidence. They allow decision makers and planners to quickly identify the best available evidence on a topic, remaining evidence gaps, as well as suitable areas to be converted into living evidence reviews (Thomas et al., 2017). For example, the Campbell evidence and gap map on people with disabilities may be helpful to inform decisions about health, social engagement and employment for people with disabilities (Saran, White, & Kuper, 2020) in the aftermath of COVID‐19 stringency measures.

**Figure 1:**
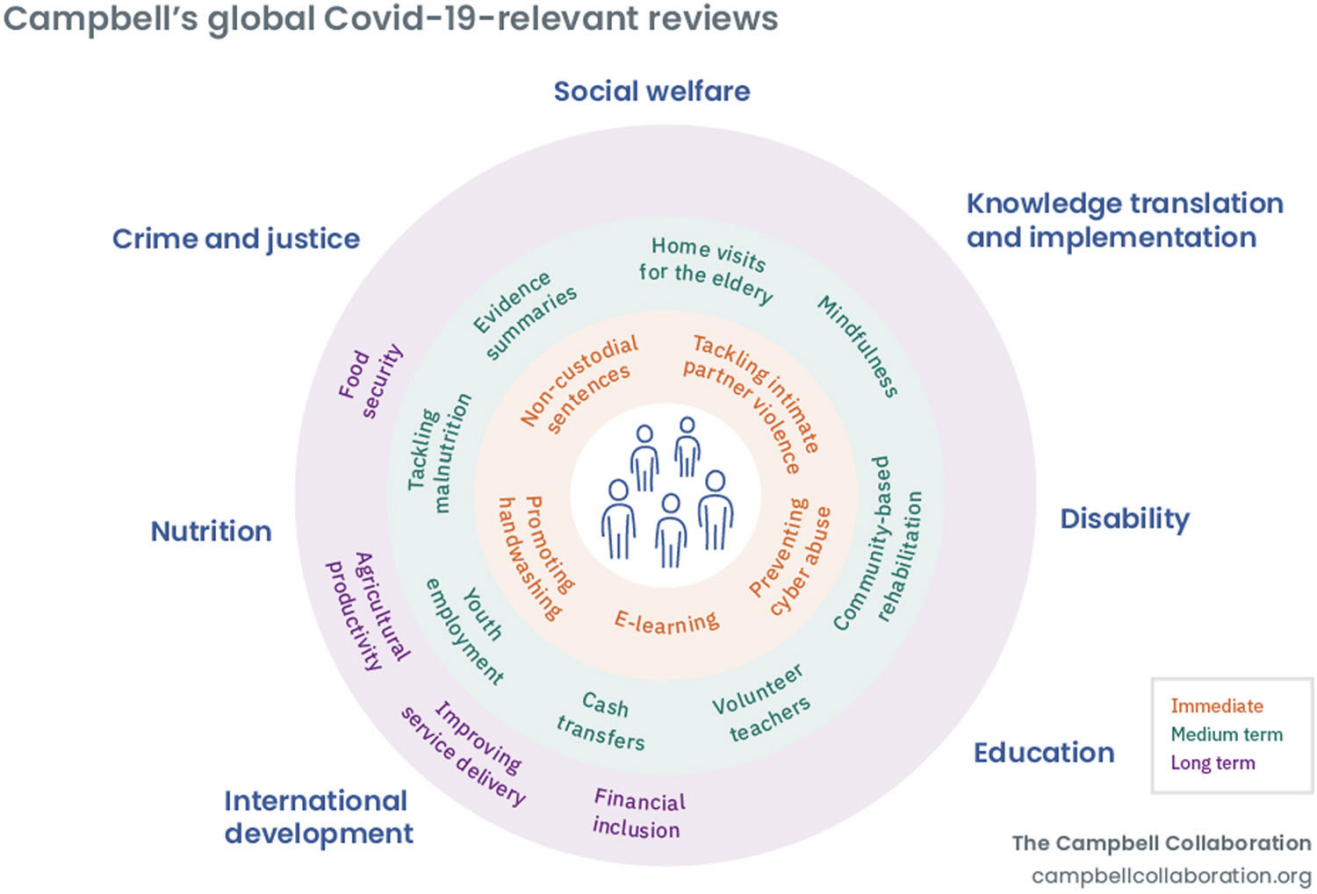
Campbell Collaboration relevance to COVID‐19 social and economic response. COVID‐19, coronavirus disease 2019

With this editorial, we provide a virtual issue of 50 Campbell systematic reviews to inform the social and economic response to COVID‐19 (Figure 1).

Some reviews have immediate relevance, including how to promote handwashing (De Buck et al., 2017), distribute cash in emergency settings, provide nutrition outreach, intervene for the safety of women and children and implement evidence‐based policing.

Lockdown measures put pressure on families. We can learn from the large number of reviews on family functioning such as promoting the well‐being of children exposed to intimate partner violence (Latzman, Casanueva, Brinton, & Forman‐Hoffman, 2019). Reviews provide guidance to support vulnerable populations including the elderly, and others needing assistance in daily living. Other reviews cover programmes to strengthen the social safety net, for example, in food security, cash transfers and care homes.

As economies reopen, Campbell reviews offer ideas on how best to get people back to work, including labour activation measures such as youth employment (Kluve et al., 2017), promoting entrepreneurship and providing vocational training. With global shutdowns in food processing plants and agriculture, we need to increase food production and availability through transport, improving retail access and outreach to difficult‐to‐reach areas such as urban slums. Campbell reviews highlight the effects of technological support for farmers, training and contract farming.

Campbell reviews inform how to restructure government services such as schools, community services and prisons to support continued social distancing. New evidence syntheses are needed in some areas to answer questions directly related to COVID‐19 policies; for example, evidence on the impacts of reopening of schools on disease burden, learning and achievement and family well‐being would be most helpful. Reviews provide evidence on alternatives to prison like noncustodial sentences (Villettaz, Gillieron, & Killias, 2015), noncustodial employment programmes and court diversion programmes to keep youth out of the justice system.

The Campbell response to COVID‐19 has included the following six main strategies to date.

1. Partnership with Evidence Aid to produce COVID‐19‐relevant summaries of Campbell systematic reviews (Evidence Aid Coronavirus COVID‐19, 2020).
2. Highlighting COVID‐19‐relevant Campbell reviews with blogs and editorials.
3. Partnership with the COVID‐END network to coordinate evidence synthesis initiatives.
4. Fast track editorial process for COVID‐19 relevant articles.
5. Development of methods to register rapid systematic reviews, followed by living reviews to address high‐priority questions with rapidly emerging evidence‐bases (ongoing).
6. Initiatives within practitioner and policy communities, such as priority‐setting, webinars and training.

*Campbell Systematic Reviews* welcomes registration of new reviews, with a fast‐track editorial process, to inform the global COVID‐19 social and economic response. Our methodological standards protect against bias and potentially misleading findings. Registration with Campbell protects against research waste since titles and protocols are publicly available and searchable.

As the world continues to respond to the COVID‐19 crisis, the policy community needs rigorous evidence on options and alternatives. Evidence from Campbell systematic reviews shows what is known on social and economic policies and programmes. Reviews identify the uncertainties to address via policy experiments, pilot tests and trials. And they identify questions to be answered with further evidence synthesis or primary research. Donald Campbell's vision of an Experimenting Society (Campbell, 1991), which conducts and learns from policy experiments, is needed now more than ever.
